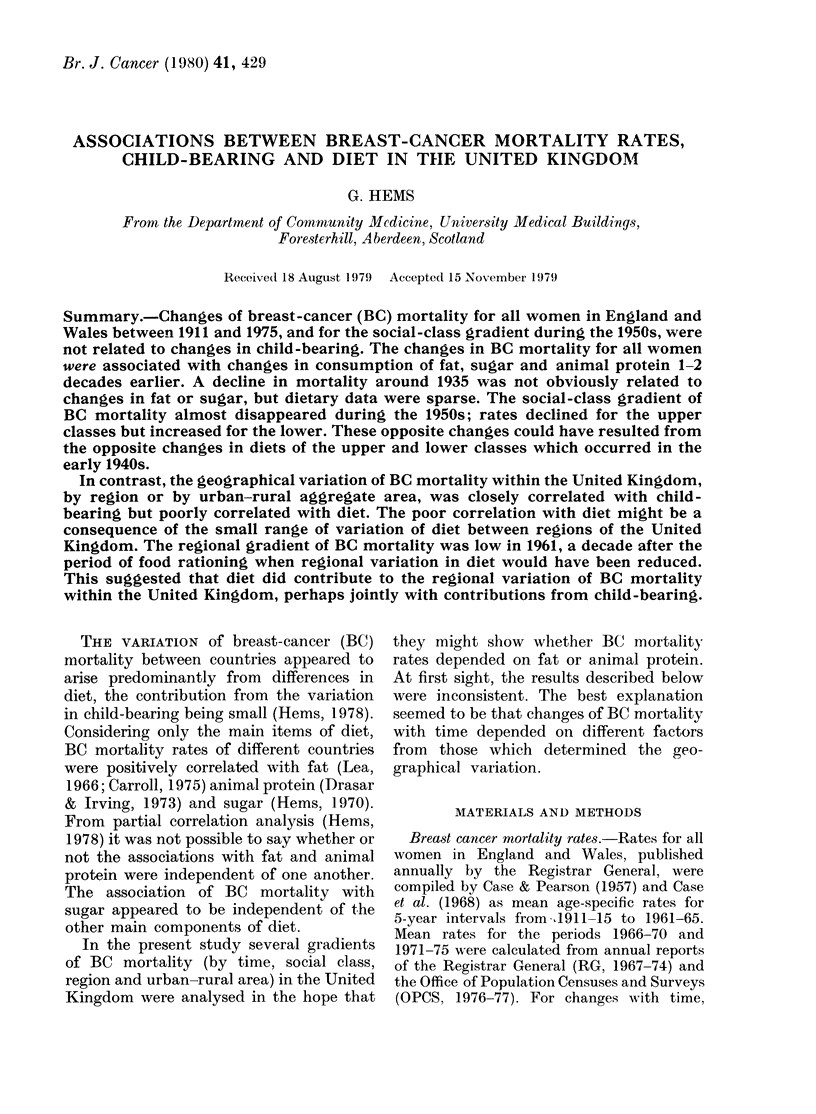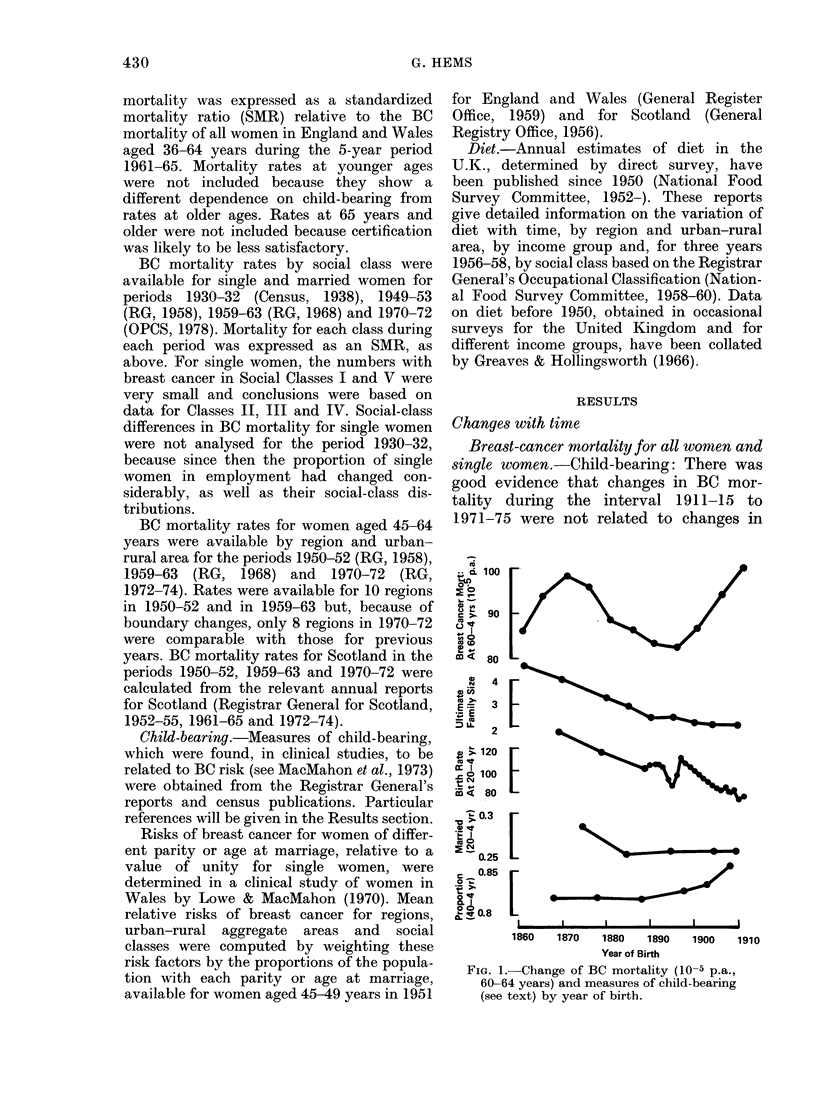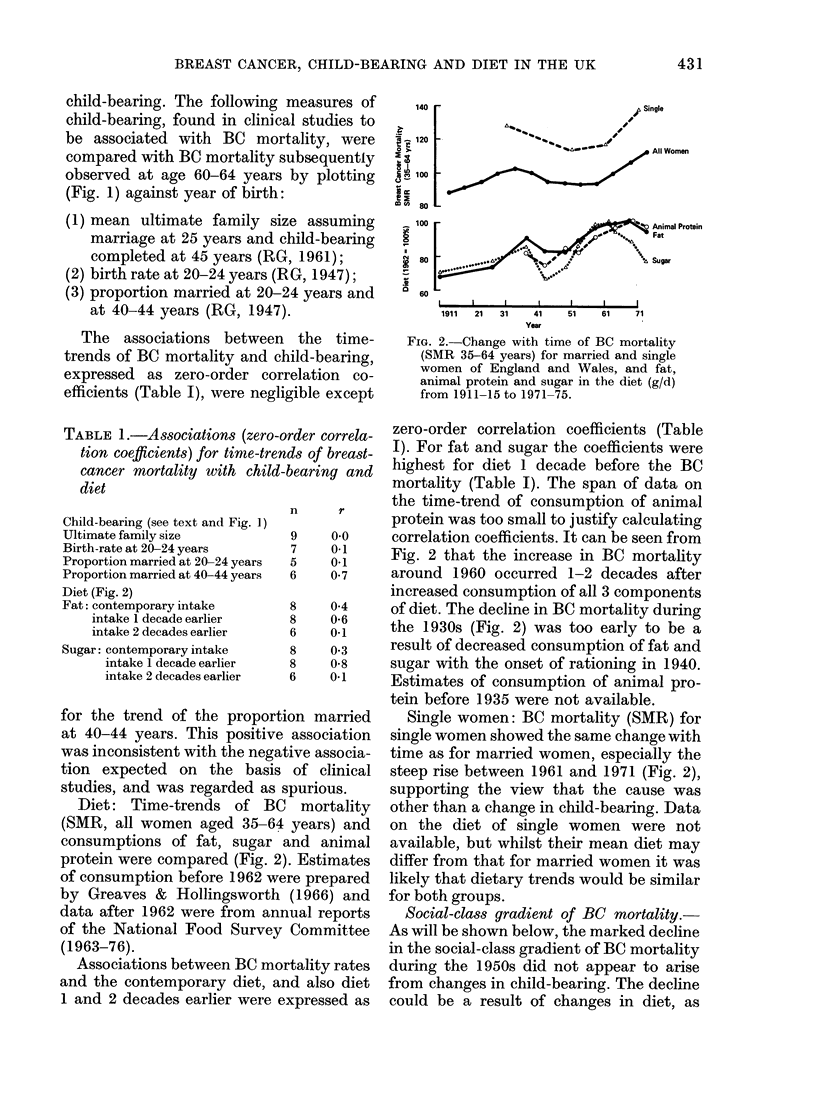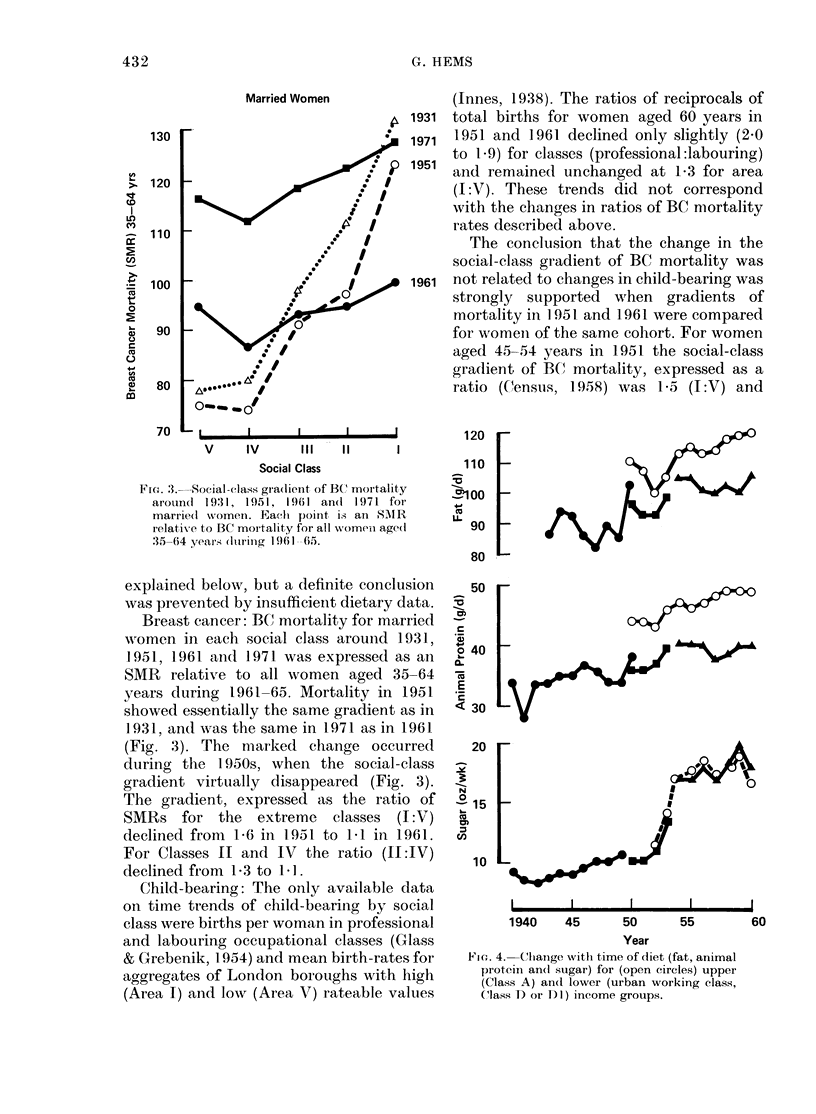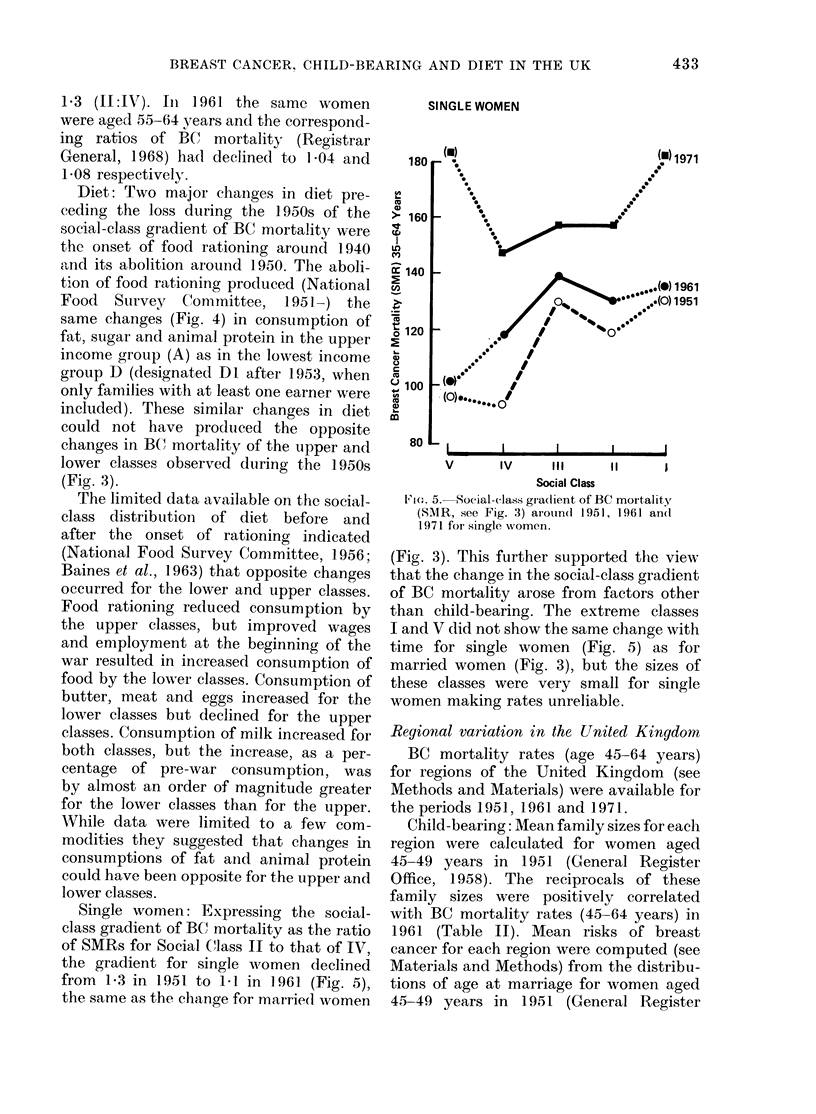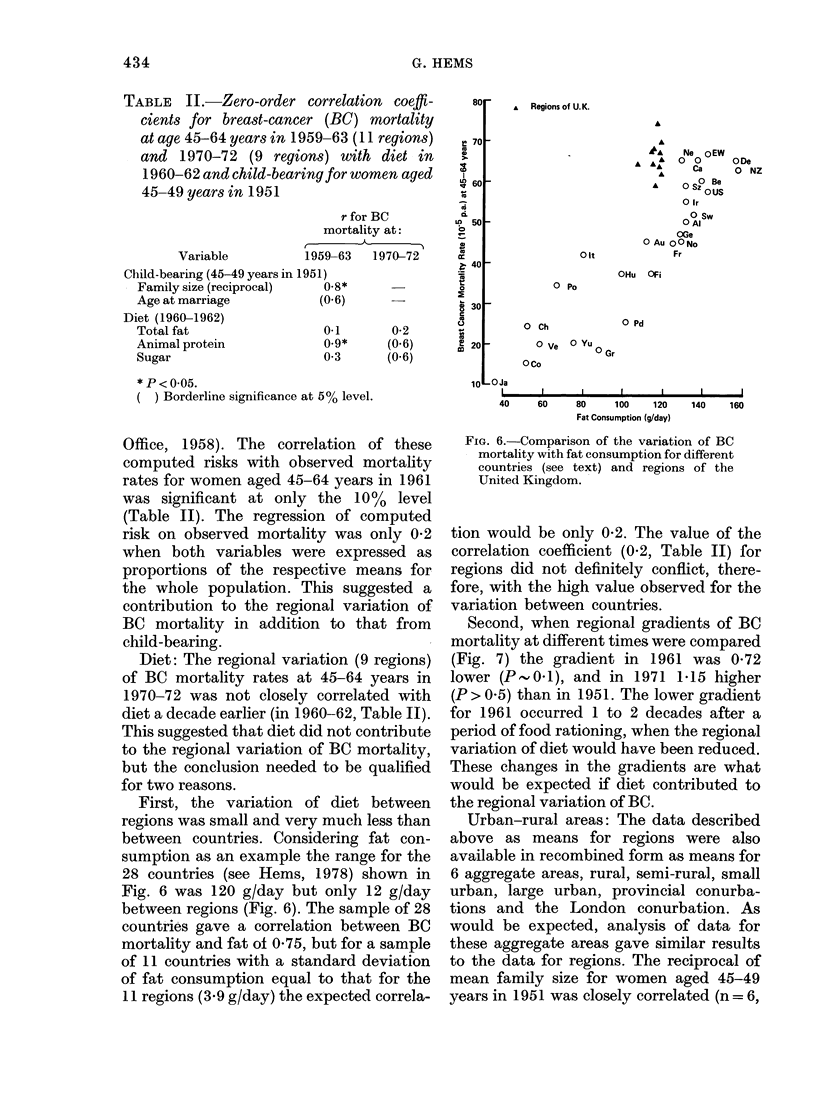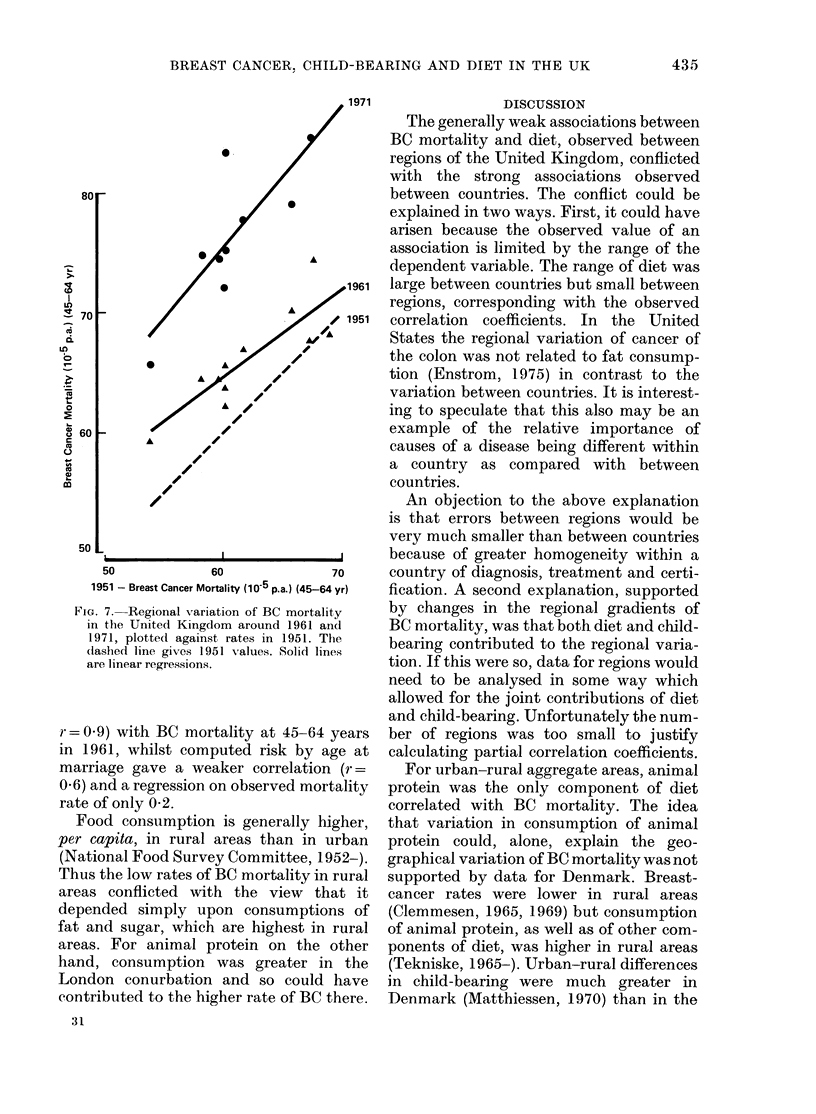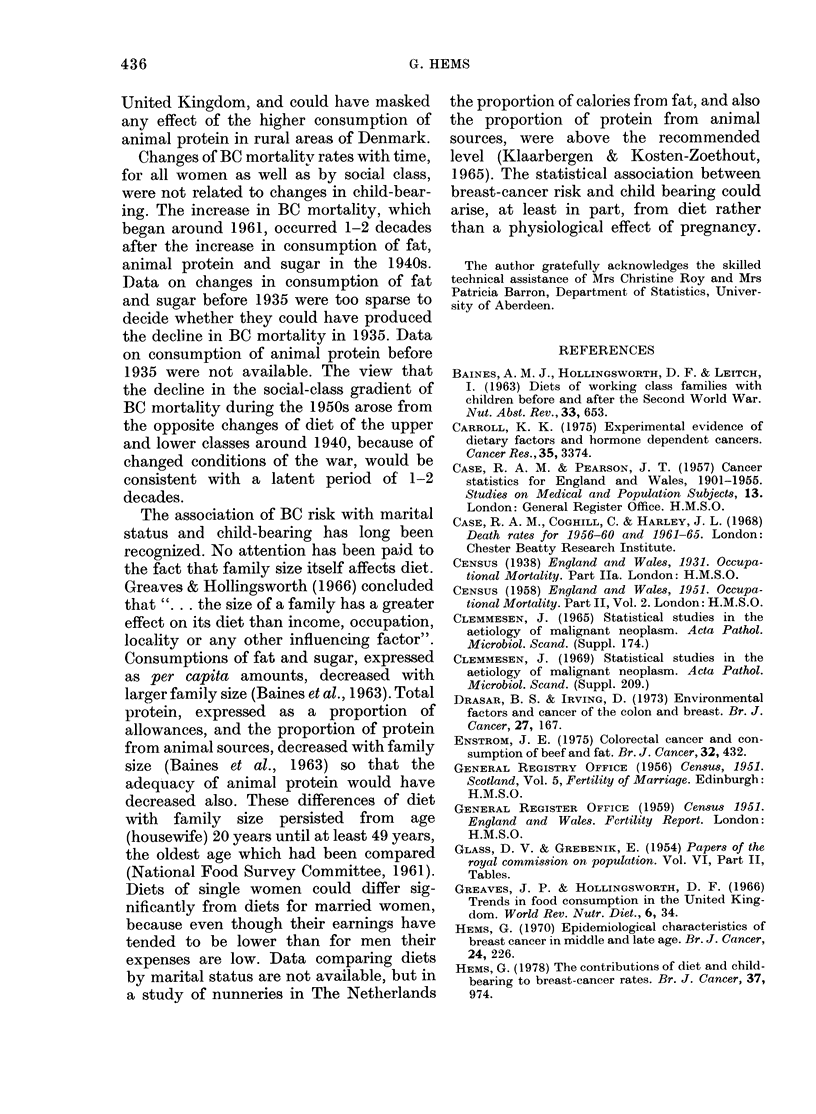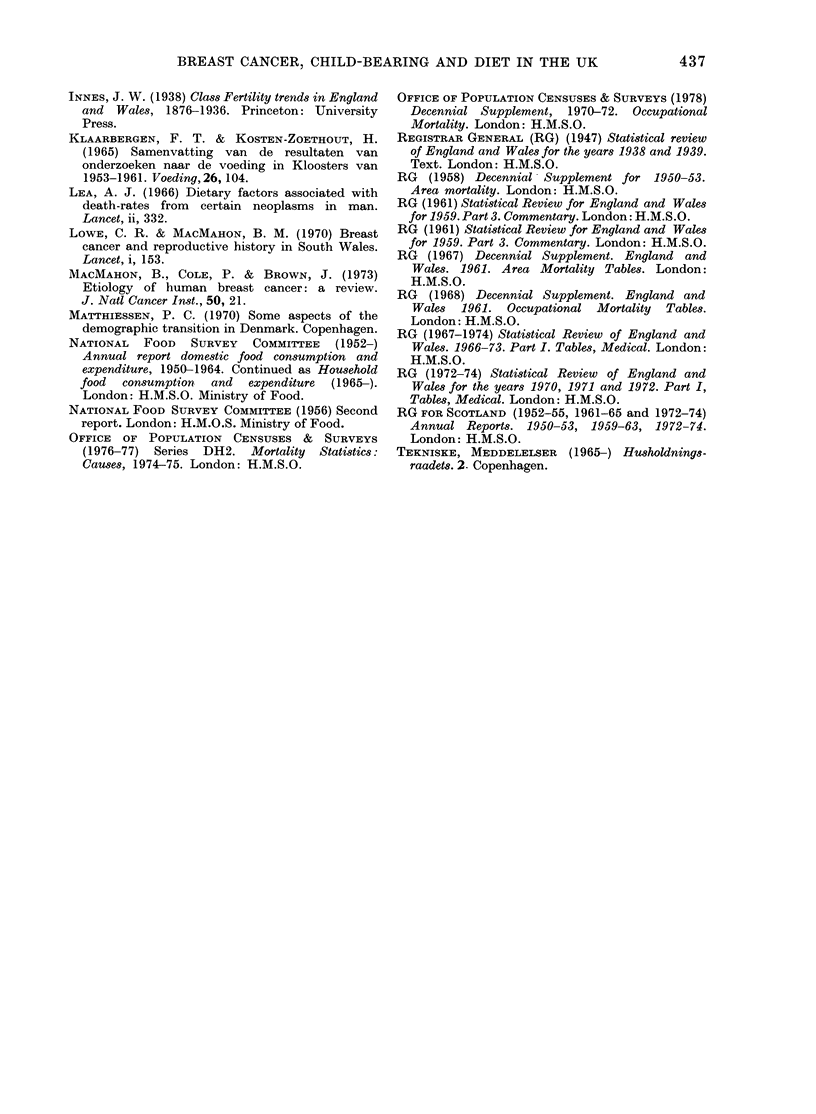# Associations between breast-cancer mortality rates, child-bearing and diet in the United Kingdom.

**DOI:** 10.1038/bjc.1980.67

**Published:** 1980-03

**Authors:** G. Hems

## Abstract

Changes of breast-cancer (BC) mortality for all women in England and Wales between 1911 and 1975, and for the social-class gradient during the 1950s, were not related to changes in child-bearing. The changes in BC mortality for all women were associated with changes in consumption of fat, sugar and animal protein 1-2 decades earlier. A decline in mortality around 1935 was not obviously related to changes in fat or sugar, but dietary data were sparse. The social-class gradient of BC mortality almost disappeared during the 1950s; rates declined for the upper classes but increased for the lower. These opposite changes could have resulted from the opposite changes in diets of the upper and lower classes which occurred in the early 1940s. In contrast, the geographical variation of BC mortality within the United Kingdom, by region or by urban-rural aggregate area, was closely correlated with child-bearing but poorly correlated with diet. The poor correlation with diet might be a consequence of the small range of variation of diet between regions of the United Kingdom. The regional gradient of BC mortality was low in 1961, a decade after the period of food rationing when regional variation in diet would have been reduced. This suggested that diet did contribute to the regional variation of BC mortality within the United Kingdom, perhaps jointly with contributions from child-bearing.


*Br. J. Cancer* (1980) **41**, 429

### ASSOCIATIONS BETWEEN BREAST-CANCER MORTALITY RATES, CHILD-BEARING AND DIET IN THE UNITED KINGDOM

G. HEMS

*From the Department of Community Medicine, University Medical Buildings, Foresterhill, Aberdeen, Scotland*

Received 18 August 1979 Accepted 15 November 1979

**Summary.—Changes of breast-cancer (BC) mortality for all women in England and Wales between 1911 and 1975, and for the social-class gradient during the 1950s, were not related to changes in child-bearing. The changes in BC mortality for all women *were* associated with changes in consumption of fat, sugar and animal protein 1–2 decades earlier. A decline in mortality around 1935 was not obviously related to changes in fat or sugar, but dietary data were sparse. The social-class gradient of BC mortality almost disappeared during the 1950s; rates declined for the upper classes but increased for the lower. These opposite changes could have resulted from the opposite changes in diets of the upper and lower classes which occurred in the early 1940s.**

**In contrast, the geographical variation of BC mortality within the United Kingdom, by region or by urban–rural aggregate area, was closely correlated with child-bearing but poorly correlated with diet. The poor correlation with diet might be a consequence of the small range of variation of diet between regions of the United Kingdom. The regional gradient of BC mortality was low in 1961, a decade after the period of food rationing when regional variation in diet would have been reduced. This suggested that diet did contribute to the regional variation of BC mortality within the United Kingdom, perhaps jointly with contributions from child-bearing.**

THE VARIATION of breast-cancer (BC) mortality between countries appeared to arise predominantly from differences in diet, the contribution from the variation in child-bearing being small (Hems, 1978). Considering only the main items of diet, BC mortality rates of different countries were positively correlated with fat (Lea, 1966; Carroll, 1975) animal protein (Drasar & Irving, 1973) and sugar (Hems, 1970). From partial correlation analysis (Hems, 1978) it was not possible to say whether or not the associations with fat and animal protein were independent of one another. The association of BC mortality with sugar appeared to be independent of the other main components of diet.

In the present study several gradients of BC mortality (by time, social class, region and urban–rural area) in the United Kingdom were analysed in the hope that they might show whether BC mortality rates depended on fat or animal protein. At first sight, the results described below were inconsistent. The best explanation seemed to be that changes of BC mortality with time depended on different factors from those which determined the geographical variation.

### MATERIALS AND METHODS

*Breast cancer mortality rates.*—Rates for all women in England and Wales, published annually by the Registrar General, were compiled by Case & Pearson (1957) and Case *et al.* (1968) as mean age-specific rates for 5-year intervals from 1911–15 to 1961–65. Mean rates for the periods 1966–70 and 1971–75 were calculated from annual reports of the Registrar General (RG, 1967–74) and the Office of Population Censuses and Surveys (OPCS, 1976–77). For changes with time, mortality was expressed as a standardized mortality ratio (SMR) relative to the BC mortality of all women in England and Wales aged 36–64 years during the 5-year period 1961–65. Mortality rates at younger ages were not included because they show a different dependence on child-bearing from rates at older ages. Rates at 65 years and older were not included because certification was likely to be less satisfactory.

BC mortality rates by social class were available for single and married women for periods 1930–32 (Census, 1938), 1949–53 (RG, 1958), 1959–63 (RG, 1968) and 1970–72 (OPCS, 1978). Mortality for each class during each period was expressed as an SMR, as above. For single women, the numbers with breast cancer in Social Classes I and V were very small and conclusions were based on data for Classes II, III and IV. Social-class differences in BC mortality for single women were not analysed for the period 1930–32, because since then the proportion of single women in employment had changed considerably, as well as their social-class distributions.

BC mortality rates for women aged 45–64 years were available by region and urban–rural area for the periods 1950–52 (RG, 1958), 1959–63 (RG, 1968) and 1970–72 (RG, 1972–74). Rates were available for 10 regions in 1950–52 and in 1959–63 but, because of boundary changes, only 8 regions in 1970–72 were comparable with those for previous years. BC mortality rates for Scotland in the periods 1950–52, 1959–63 and 1970–72 were calculated from the relevant annual reports for Scotland (Registrar General for Scotland, 1952–55, 1961–65 and 1972–74).

*Child-bearing.*—Measures of child-bearing, which were found, in clinical studies, to be related to BC risk (see MacMahon *et al.*, 1973) were obtained from the Registrar General's reports and census publications. Particular references will be given in the Results section.

Risks of breast cancer for women of different parity or age at marriage, relative to a value of unity for single women, were determined in a clinical study of women in Wales by Lowe & MacMahon (1970). Mean relative risks of breast cancer for regions, urban–rural aggregate areas and social classes were computed by weighting these risk factors by the proportions of the population with each parity or age at marriage, available for women aged 45–49 years in 1951 for England and Wales (General Register Office, 1959) and for Scotland (General Registry Office, 1956).

*Diet.*—Annual estimates of diet in the U.K., determined by direct survey, have been published since 1950 (National Food Survey Committee, 1952–). These reports give detailed information on the variation of diet with time, by region and urban–rural area, by income group and, for three years 1956–58, by social class based on the Registrar General's Occupational Classification (National Food Survey Committee, 1958–60). Data on diet before 1950, obtained in occasional surveys for the United Kingdom and for different income groups, have been collated by Greaves & Hollingsworth (1966).

### RESULTS

### *Changes with time*

*Breast-cancer mortality for all women and single women.*—Child-bearing: There was good evidence that changes in BC mortality during the interval 1911–15 to 1971–75 were not related to changes in

FIG. 1.—Change of BC mortality ($10^{-5}$ p.a., 60–64 years) and measures of child-bearing (see text) by year of birth.

child-bearing. The following measures of child-bearing, found in clinical studies to be associated with BC mortality, were compared with BC mortality subsequently observed at age 60–64 years by plotting (Fig. 1) against year of birth:

(1) mean ultimate family size assuming marriage at 25 years and child-bearing completed at 45 years (RG, 1961);
(2) birth rate at 20–24 years (RG, 1947);
(3) proportion married at 20–24 years and at 40–44 years (RG, 1947).

The associations between the time-trends of BC mortality and child-bearing, expressed as zero-order correlation coefficients (Table I), were negligible except for the trend of the proportion married at 40–44 years. This positive association was inconsistent with the negative association expected on the basis of clinical studies, and was regarded as spurious.

Diet: Time-trends of BC mortality (SMR, all women aged 35–64 years) and consumptions of fat, sugar and animal protein were compared (Fig. 2). Estimates of consumption before 1962 were prepared by Greaves & Hollingsworth (1966) and data after 1962 were from annual reports of the National Food Survey Committee (1963–76).

Associations between BC mortality rates and the contemporary diet, and also diet 1 and 2 decades earlier were expressed as zero-order correlation coefficients (Table I). For fat and sugar the coefficients were highest for diet 1 decade before the BC mortality (Table I). The span of data on the time-trend of consumption of animal protein was too small to justify calculating correlation coefficients. It can be seen from Fig. 2 that the increase in BC mortality around 1960 occurred 1–2 decades after increased consumption of all 3 components of diet. The decline in BC mortality during the 1930s (Fig. 2) was too early to be a result of decreased consumption of fat and sugar with the onset of rationing in 1940. Estimates of consumption of animal protein before 1935 were not available.

TABLE 1.—*Associations (zero-order correlation coefficients) for time-trends of breast-cancer mortality with child-bearing and diet*

| | n | r |
|---|---|---|
| Child-bearing (see text and Fig. 1) | | |
| Ultimate family size | 9 | 0·0 |
| Birth-rate at 20–24 years | 7 | 0·1 |
| Proportion married at 20–24 years | 5 | 0·1 |
| Proportion married at 40–44 years | 6 | 0·7 |
| Diet (Fig. 2) | | |
| Fat: contemporary intake | 8 | 0·4 |
| intake 1 decade earlier | 8 | 0·6 |
| intake 2 decades earlier | 6 | 0·1 |
| Sugar: contemporary intake | 8 | 0·3 |
| intake 1 decade earlier | 8 | 0·8 |
| intake 2 decades earlier | 6 | 0·1 |

FIG. 2.—Change with time of BC mortality (SMR 35–64 years) for married and single women of England and Wales, and fat, animal protein and sugar in the diet (g/d) from 1911–15 to 1971–75.

Single women: BC mortality (SMR) for single women showed the same change with time as for married women, especially the steep rise between 1961 and 1971 (Fig. 2), supporting the view that the cause was other than a change in child-bearing. Data on the diet of single women were not available, but whilst their mean diet may differ from that for married women it was likely that dietary trends would be similar for both groups.

*Social-class gradient of BC mortality.*—As will be shown below, the marked decline in the social-class gradient of BC mortality during the 1950s did not appear to arise from changes in child-bearing. The decline could be a result of changes in diet, as

Fig. 3.—Social-class gradient of BC mortality around 1931, 1951, 1961 and 1971 for married women. Each point is an SMR relative to BC mortality for all women aged 35–64 years during 1961–65.

explained below, but a definite conclusion was prevented by insufficient dietary data.

Breast cancer: BC mortality for married women in each social class around 1931, 1951, 1961 and 1971 was expressed as an SMR relative to all women aged 35–64 years during 1961–65. Mortality in 1951 showed essentially the same gradient as in 1931, and was the same in 1971 as in 1961 (Fig. 3). The marked change occurred during the 1950s, when the social-class gradient virtually disappeared (Fig. 3). The gradient, expressed as the ratio of SMRs for the extreme classes (I:V) declined from 1·6 in 1951 to 1·1 in 1961. For Classes II and IV the ratio (II:IV) declined from 1·3 to 1·1.

Child-bearing: The only available data on time trends of child-bearing by social class were births per woman in professional and labouring occupational classes (Glass & Grebenik, 1954) and mean birth-rates for aggregates of London boroughs with high (Area I) and low (Area V) rateable values (Innes, 1938). The ratios of reciprocals of total births for women aged 60 years in 1951 and 1961 declined only slightly (2·0 to 1·9) for classes (professional:labouring) and remained unchanged at 1·3 for area (I:V). These trends did not correspond with the changes in ratios of BC mortality rates described above.

The conclusion that the change in the social-class gradient of BC mortality was not related to changes in child-bearing was strongly supported when gradients of mortality in 1951 and 1961 were compared for women of the same cohort. For women aged 45–54 years in 1951 the social-class gradient of BC mortality, expressed as a ratio (Census, 1958) was 1·5 (I:V) and

Fig. 4.—Change with time of diet (fat, animal protein and sugar) for (open circles) upper (Class A) and lower (urban working class, Class D or D1) income groups.

1·3 (II:IV). In 1961 the same women were aged 55–64 years and the corresponding ratios of BC mortality (Registrar General, 1968) had declined to 1·04 and 1·08 respectively.

Diet: Two major changes in diet preceding the loss during the 1950s of the social-class gradient of BC mortality were the onset of food rationing around 1940 and its abolition around 1950. The abolition of food rationing produced (National Food Survey Committee, 1951–) the same changes (Fig. 4) in consumption of fat, sugar and animal protein in the upper income group (A) as in the lowest income group D (designated D1 after 1953, when only families with at least one earner were included). These similar changes in diet could not have produced the opposite changes in BC mortality of the upper and lower classes observed during the 1950s (Fig. 3).

The limited data available on the social-class distribution of diet before and after the onset of rationing indicated (National Food Survey Committee, 1956; Baines *et al.*, 1963) that opposite changes occurred for the lower and upper classes. Food rationing reduced consumption by the upper classes, but improved wages and employment at the beginning of the war resulted in increased consumption of food by the lower classes. Consumption of butter, meat and eggs increased for the lower classes but declined for the upper classes. Consumption of milk increased for both classes, but the increase, as a percentage of pre-war consumption, was by almost an order of magnitude greater for the lower classes than for the upper. While data were limited to a few commodities they suggested that changes in consumptions of fat and animal protein could have been opposite for the upper and lower classes.

Single women: Expressing the social-class gradient of BC mortality as the ratio of SMRs for Social Class II to that of IV, the gradient for single women declined from 1·3 in 1951 to 1·1 in 1961 (Fig. 5), the same as the change for married women

FIG. 5.—Social-class gradient of BC mortality (SMR, see Fig. 3) around 1951, 1961 and 1971 for single women.

(Fig. 3). This further supported the view that the change in the social-class gradient of BC mortality arose from factors other than child-bearing. The extreme classes I and V did not show the same change with time for single women (Fig. 5) as for married women (Fig. 3), but the sizes of these classes were very small for single women making rates unreliable.

### *Regional variation in the United Kingdom*

BC mortality rates (age 45–64 years) for regions of the United Kingdom (see Methods and Materials) were available for the periods 1951, 1961 and 1971.

Child-bearing: Mean family sizes for each region were calculated for women aged 45–49 years in 1951 (General Register Office, 1958). The reciprocals of these family sizes were positively correlated with BC mortality rates (45–64 years) in 1961 (Table II). Mean risks of breast cancer for each region were computed (see Materials and Methods) from the distributions of age at marriage for women aged 45–49 years in 1951 (General Register

TABLE II.—*Zero-order correlation coefficients for breast-cancer (BC) mortality at age* 45–64 *years in* 1959–63 (11 *regions*) *and* 1970–72 (9 *regions*) *with diet in* 1960–62 *and child-bearing for women aged* 45–49 *years in* 1951

| Variable | r for BC mortality at: 1959–63 | 1970–72 |
|---|---|---|
| Child-bearing (45–49 years in 1951) | | |
| Family size (reciprocal) | 0·8* | — |
| Age at marriage | (0·6) | — |
| Diet (1960–1962) | | |
| Total fat | 0·1 | 0·2 |
| Animal protein | 0·9* | (0·6) |
| Sugar | 0·3 | (0·6) |

* $P < 0{\cdot}05$.
( ) Borderline significance at 5% level.

FIG. 6.—Comparison of the variation of BC mortality with fat consumption for different countries (see text) and regions of the United Kingdom.

Office, 1958). The correlation of these computed risks with observed mortality rates for women aged 45–64 years in 1961 was significant at only the 10% level (Table II). The regression of computed risk on observed mortality was only 0·2 when both variables were expressed as proportions of the respective means for the whole population. This suggested a contribution to the regional variation of BC mortality in addition to that from child-bearing.

Diet: The regional variation (9 regions) of BC mortality rates at 45–64 years in 1970–72 was not closely correlated with diet a decade earlier (in 1960–62, Table II). This suggested that diet did not contribute to the regional variation of BC mortality, but the conclusion needed to be qualified for two reasons.

First, the variation of diet between regions was small and very much less than between countries. Considering fat consumption as an example the range for the 28 countries (see Hems, 1978) shown in Fig. 6 was 120 g/day but only 12 g/day between regions (Fig. 6). The sample of 28 countries gave a correlation between BC mortality and fat of 0·75, but for a sample of 11 countries with a standard deviation of fat consumption equal to that for the 11 regions (3·9 g/day) the expected correlation would be only 0·2. The value of the correlation coefficient (0·2, Table II) for regions did not definitely conflict, therefore, with the high value observed for the variation between countries.

Second, when regional gradients of BC mortality at different times were compared (Fig. 7) the gradient in 1961 was 0·72 lower ($P \sim 0{\cdot}1$), and in 1971 1·15 higher ($P > 0{\cdot}5$) than in 1951. The lower gradient for 1961 occurred 1 to 2 decades after a period of food rationing, when the regional variation of diet would have been reduced. These changes in the gradients are what would be expected if diet contributed to the regional variation of BC.

Urban–rural areas: The data described above as means for regions were also available in recombined form as means for 6 aggregate areas, rural, semi-rural, small urban, large urban, provincial conurbations and the London conurbation. As would be expected, analysis of data for these aggregate areas gave similar results to the data for regions. The reciprocal of mean family size for women aged 45–49 years in 1951 was closely correlated (n = 6,

FIG. 7.—Regional variation of BC mortality in the United Kingdom around 1961 and 1971, plotted against rates in 1951. The dashed line gives 1951 values. Solid lines are linear regressions.

$r = 0{\cdot}9$) with BC mortality at 45–64 years in 1961, whilst computed risk by age at marriage gave a weaker correlation ($r = 0{\cdot}6$) and a regression on observed mortality rate of only 0·2.

Food consumption is generally higher, *per capita*, in rural areas than in urban (National Food Survey Committee, 1952–). Thus the low rates of BC mortality in rural areas conflicted with the view that it depended simply upon consumptions of fat and sugar, which are highest in rural areas. For animal protein on the other hand, consumption was greater in the London conurbation and so could have contributed to the higher rate of BC there.

DISCUSSION

The generally weak associations between BC mortality and diet, observed between regions of the United Kingdom, conflicted with the strong associations observed between countries. The conflict could be explained in two ways. First, it could have arisen because the observed value of an association is limited by the range of the dependent variable. The range of diet was large between countries but small between regions, corresponding with the observed correlation coefficients. In the United States the regional variation of cancer of the colon was not related to fat consumption (Enstrom, 1975) in contrast to the variation between countries. It is interesting to speculate that this also may be an example of the relative importance of causes of a disease being different within a country as compared with between countries.

An objection to the above explanation is that errors between regions would be very much smaller than between countries because of greater homogeneity within a country of diagnosis, treatment and certification. A second explanation, supported by changes in the regional gradients of BC mortality, was that both diet and child-bearing contributed to the regional variation. If this were so, data for regions would need to be analysed in some way which allowed for the joint contributions of diet and child-bearing. Unfortunately the number of regions was too small to justify calculating partial correlation coefficients.

For urban–rural aggregate areas, animal protein was the only component of diet correlated with BC mortality. The idea that variation in consumption of animal protein could, alone, explain the geographical variation of BC mortality was not supported by data for Denmark. Breast-cancer rates were lower in rural areas (Clemmesen, 1965, 1969) but consumption of animal protein, as well as of other components of diet, was higher in rural areas (Tekniske, 1965–). Urban–rural differences in child-bearing were much greater in Denmark (Matthiessen, 1970) than in the

United Kingdom, and could have masked any effect of the higher consumption of animal protein in rural areas of Denmark.

Changes of BC mortality rates with time, for all women as well as by social class, were not related to changes in child-bearing. The increase in BC mortality, which began around 1961, occurred 1–2 decades after the increase in consumption of fat, animal protein and sugar in the 1940s. Data on changes in consumption of fat and sugar before 1935 were too sparse to decide whether they could have produced the decline in BC mortality in 1935. Data on consumption of animal protein before 1935 were not available. The view that the decline in the social-class gradient of BC mortality during the 1950s arose from the opposite changes of diet of the upper and lower classes around 1940, because of changed conditions of the war, would be consistent with a latent period of 1–2 decades.

The association of BC risk with marital status and child-bearing has long been recognized. No attention has been paid to the fact that family size itself affects diet. Greaves & Hollingsworth (1966) concluded that "... the size of a family has a greater effect on its diet than income, occupation, locality or any other influencing factor". Consumptions of fat and sugar, expressed as *per capita* amounts, decreased with larger family size (Baines *et al.*, 1963). Total protein, expressed as a proportion of allowances, and the proportion of protein from animal sources, decreased with family size (Baines *et al.*, 1963) so that the adequacy of animal protein would have decreased also. These differences of diet with family size persisted from age (housewife) 20 years until at least 49 years, the oldest age which had been compared (National Food Survey Committee, 1961). Diets of single women could differ significantly from diets for married women, because even though their earnings have tended to be lower than for men their expenses are low. Data comparing diets by marital status are not available, but in a study of nunneries in The Netherlands the proportion of calories from fat, and also the proportion of protein from animal sources, were above the recommended level (Klaarbergen & Kosten-Zoethout, 1965). The statistical association between breast-cancer risk and child bearing could arise, at least in part, from diet rather than a physiological effect of pregnancy.

The author gratefully acknowledges the skilled technical assistance of Mrs Christine Roy and Mrs Patricia Barron, Department of Statistics, University of Aberdeen.

### REFERENCES

Baines, A. M. J., Hollingsworth, D. F. & Leitch, I. (1963) Diets of working class families with children before and after the Second World War. *Nut. Abst. Rev.*, **33**, 653.

Carroll, K. K. (1975) Experimental evidence of dietary factors and hormone dependent cancers. *Cancer Res.*, **35**, 3374.

Case, R. A. M. & Pearson, J. T. (1957) Cancer statistics for England and Wales, 1901–1955. *Studies on Medical and Population Subjects*, **13.** London: General Register Office. H.M.S.O.

Case, R. A. M., Coghill, C. & Harley, J. L. (1968) *Death rates for 1956–60 and 1961–65.* London: Chester Beatty Research Institute.

Census (1938) *England and Wales, 1931. Occupational Mortality.* Part IIa. London: H.M.S.O.

Census (1958) *England and Wales, 1951. Occupational Mortality.* Part II, Vol. 2. London: H.M.S.O.

Clemmesen, J. (1965) Statistical studies in the aetiology of malignant neoplasm. *Acta Pathol. Microbiol. Scand.* (Suppl. 174.)

Clemmesen, J. (1969) Statistical studies in the aetiology of malignant neoplasm. *Acta Pathol. Microbiol. Scand.* (Suppl. 209.)

Drasar, B. S. & Irving, D. (1973) Environmental factors and cancer of the colon and breast. *Br. J. Cancer*, **27**, 167.

Enstrom, J. E. (1975) Colorectal cancer and consumption of beef and fat. *Br. J. Cancer*, **32**, 432.

General Registry Office (1956) *Census, 1951. Scotland*, Vol. 5, *Fertility of Marriage.* Edinburgh: H.M.S.O.

General Register Office (1959) *Census 1951. England and Wales. Fertility Report.* London: H.M.S.O.

Glass, D. V. & Grebenik, E. (1954) *Papers of the royal commission on population.* Vol. VI, Part II, Tables.

Greaves, J. P. & Hollingsworth, D. F. (1966) Trends in food consumption in the United Kingdom. *World Rev. Nutr. Diet.*, **6**, 34.

Hems, G. (1970) Epidemiological characteristics of breast cancer in middle and late age. *Br. J. Cancer*, **24**, 226.

Hems, G. (1978) The contributions of diet and child-bearing to breast-cancer rates. *Br. J. Cancer*, **37**, 974.

INNES, J. W. (1938) *Class Fertility trends in England and Wales*, 1876–1936. Princeton: University Press.

KLAARBERGEN, F. T. & KOSTEN-ZOETHOUT, H. (1965) Samenvatting van de resultaten van onderzoeken naar de voeding in Kloosters van 1953–1961. *Voeding*, **26**, 104.

LEA, A. J. (1966) Dietary factors associated with death-rates from certain neoplasms in man. *Lancet*, ii, 332.

LOWE, C. R. & MACMAHON, B. M. (1970) Breast cancer and reproductive history in South Wales. *Lancet*, i, 153.

MACMAHON, B., COLE, P. & BROWN, J. (1973) Etiology of human breast cancer: a review. *J. Natl Cancer Inst.*, **50**, 21.

MATTHIESSEN, P. C. (1970) Some aspects of the demographic transition in Denmark. Copenhagen.

NATIONAL FOOD SURVEY COMMITTEE (1952–) *Annual report domestic food consumption and expenditure*, 1950–1964. Continued as *Household food consumption and expenditure* (1965–). London: H.M.S.O. Ministry of Food.

NATIONAL FOOD SURVEY COMMITTEE (1956) Second report. London: H.M.O.S. Ministry of Food.

OFFICE OF POPULATION CENSUSES & SURVEYS (1976–77) Series DH2. *Mortality Statistics: Causes*, 1974–75. London: H.M.S.O.

OFFICE OF POPULATION CENSUSES & SURVEYS (1978) *Decennial Supplement*, 1970–72. *Occupational Mortality*. London: H.M.S.O.

REGISTRAR GENERAL (RG) (1947) *Statistical review of England and Wales for the years 1938 and 1939*. Text. London: H.M.S.O.

RG (1958) *Decennial Supplement for 1950–53. Area mortality*. London: H.M.S.O.

RG (1961) *Statistical Review for England and Wales for 1959. Part 3. Commentary*. London: H.M.S.O.

RG (1961) *Statistical Review for England and Wales for 1959. Part 3. Commentary*. London: H.M.S.O.

RG (1967) *Decennial Supplement. England and Wales. 1961. Area Mortality Tables*. London: H.M.S.O.

RG (1968) *Decennial Supplement. England and Wales 1961. Occupational Mortality Tables*. London: H.M.S.O.

RG (1967–1974) *Statistical Review of England and Wales. 1966–73. Part I. Tables, Medical*. London: H.M.S.O.

RG (1972–74) *Statistical Review of England and Wales for the years 1970, 1971 and 1972. Part I, Tables, Medical*. London: H.M.S.O.

RG FOR SCOTLAND (1952–55, 1961–65 and 1972–74) *Annual Reports. 1950–53, 1959–63, 1972–74*. London: H.M.S.O.

TEKNISKE, MEDDELELSER (1965–) *Husholdningsraadets*. 2. Copenhagen.